# Effect of Bacterial Vaginosis (BV)-HIV-1 Co-existence on Maternal and Infant Health: A Secondary Data Analysis

**DOI:** 10.3389/fped.2021.544192

**Published:** 2021-03-18

**Authors:** Ngugi Mwenda, Ruth Nduati, Mathew Kosgey, Gregory Kerich

**Affiliations:** ^1^Department of Mathematics, Physics and Computing, School of Sciences and Aerospace Studies, Moi University, Eldoret, Kenya; ^2^Department of Paediatrics, University of Nairobi, Nairobi, Kenya

**Keywords:** mortality, HIV, infant, bacterial vaginosis, morbidity

## Abstract

**Background:** The lactobacillus-rich microbiome forms a defense system against infections. Babies are born sterile and acquire their microbiome from exposure to the mothers' vaginal and rectal microbiota. Bacterial vaginosis (BV), which is characterized by a deficit of the Lactobacilli genera, may predispose women and their babies to an increased frequency of illness.

**Objective:** To determine the effect of BV on HIV-infected women's post-delivery health as well as the morbidity and mortality of the exposed infant at birth, 6 months, and at 12 months of life.

**Study Design:** A retrospective cohort study was conducted using previously collected data to investigate whether there was an association between BV-HIV-1 infected mothers and subsequent infant morbidity and mortality over a 12-month period.

**Methods:** Data for this analysis were extracted from the original data set. Women were categorized into two groups according to whether they had a positive or negative laboratory-based diagnosis of BV using the Nugent method. The two groups were compared for socio-demographic characteristics, prior to the pregnancy experience in their current pregnancy outcome and at post-delivery morbidity, and for the duration of hospital stay. BV-exposed and unexposed infants were compared in terms of morbidity and mortality at birth, and in the periods between birth and 6 months, and between 6 and 12 months, respectively, based on prospectively collected data of the mother's past and present illness, and clinical examination at scheduled and unscheduled visits during the follow-up period of the original study. The generalized estimating equation (GEE) was used to analyze the longitudinally collected data. We used the Kaplan-Meier (KM) method to generate the cumulative hazard curve and compared the mortality in the first year of life between the two groups.

**Results:** In total, 365 patients were included in the study. Exposure to BV was associated with an adverse maternal condition (Relative Risk [RR], 2.45; 95% confidence interval [CI], 1.04–5.81, *P* = 0.04) and maternal hospital admission (RR, 1.99; 95% CI, 1.14–3.48, *P* = 0.02) but was not linked to any neonatal morbidity at birth. There was a higher frequency of gastro-intestinal morbidity among BV-exposed infants. At 6 months, infants of BV-exposed mothers had higher odds of bloody stool (Odds Ratio [OR], 3.08; 95% CI, 1.11–10.00, *P* = 0.04), dehydration (OR, 2.94; 95% CI, 1.44–6.37, *P* = 0.01), vomiting (OR, 1.64; 95% CI, 1.06–2.56, *P* = 0.03), and mouth ulcers (OR, 12.8; 95% CI, 2.27–241.21, *P* = 0.02). At 12 months, exposure to BV was associated with dehydration (OR, 1.81; 95% CI, 1.05–3.19, *P* = 0.03) and vomiting (OR, 1.39; 95% CI, 1.01–1.92, *P* = 0.04). KM survival analysis showed non-significant higher trends of deaths among BV-exposed infants (*P* = 0.65).

**Conclusion:** This study demonstrates differences in maternal and infant morbidity outcomes associated with exposure to BV. Further research is required to determine whether treatment for maternal BV mitigates maternal and infant morbidity.

## 1. Introduction

Bacterial vaginosis (BV), also referred to as vaginal dysbiosis, is characterized by altered vaginal biota, which typically has a deficit of *Lactobacillus bifidus* and an overgrowth of anaerobic polybacteria that include *Clostridiales, Fusobacterium nucleatum, Gardnerella vaginalis, Mycoplasma hominis*, and *Bacteroides urealyticus*, among others. BV may co-exist with other anerobic bacteria, such as *Streptococcus, Staphylococcus*, and *Enterobacteriaceae*, typically found in the lower genital tract (1).

BV has been shown to be a risk factor for pre-term births (2). A pre-term birth is defined as a delivery before 37 completed weeks of gestation either from labor induced due to a fetal condition, from spontaneous labor with intact membranes, or following a premature rupture of membranes (PROM). The latter is more common among Black women and is attributed to genital tract infection in both human immunodeficiency virus (HIV) seronegative and seropositive women (2–4). Prematurity in the presence of BV is additionally associated with a low birth weight (LBW) (5, 6). It is estimated that annually, about 13 million babies are born before 37 completed weeks of gestation; BV-related deaths account for 75% of the total perinatal deaths and 27% of the total neonatal mortality cases. This makes BV a condition of significant public health importance and requires further evaluation (2, 3, 7, 8).

Infant-related adverse effects of BV exposure are not limited to causing pre-term births, as illustrated in a retrospective review of 12,340 mother–baby pairs identified from Washington State medical records, which found that BV-exposed babies were more likely to have meconium staining at birth, which is indicative of *in-utero* stress. In the same study, independent of meconium staining and chorioamnionitis, BV-exposed term babies were at an increased risk of respiratory distress requiring ventilatory support, admission to a neonatal intensive care unit (NICU), and neonatal sepsis (9). There are limited data on whether BV has an effect on infant morbidity and mortality beyond the immediate newborn period.

A healthy vaginal microbiome has a preponderance of the Lactobacillus genera (2). These anaerobic bacteria contribute to an acidic environment by fermenting sugars that produce lactic acid. The acidic environment helps protect the uterus from ascending infections, such as sexually transmitted diseases and urinary tract infections. The same principle applies for other body surfaces including the gastrointestinal system. There is enough evidence showing that good bacteria in women lower the risk of infections (10) and HIV (11). Although there are no known direct causes of BV, a large body of literature reports an increased probability of infection due to sexual activity. Two studies conducted in Kenya provide evidence for this with the observation that 90% of sexually inactive women had normal vaginal flora (12, 13), whereas Bukusi et al., reported a high BV prevalence of 44% among married couples (14). BV, however, does not meet the threshold of being classified as a sexually transmitted infection because the male partner does not experience any illness and there is lack of a single causative agent.

BV often co-exists with sexually transmitted infections. A meta-analysis of 37,000 HIV-negative women from sub-Saharan Africa found that >50% of women with different STDs had co-existing BV and there was a significant correlation between BV and herpes simplex type 2 (HSV-2) and Trichomonas (15). BV increases women's risk of acquiring HIV (16, 17), and once infected, it is associated with the increased genital shedding of HIV, a risk factor for intrapartum HIV transmission (18). BV-HIV co-infection is associated with a 3-fold increased risk of *in-utero* mother-to-child transmission of HIV after adjusting for maternal HIV-1 viral load (19).

African women carry a disproportionate burden of BV. In the meta-analysis published by Torrone et al., the prevalence of BV among HIV-negative women in sub-Saharan Africa was >40% higher in the high-risk groups, compared to women recruited at community level or at health clinics, but the difference was not significant (49.5 vs. 35.2%) (15). An equally high prevalence of BV has been described among HIV-infected women; 46% in a high-risk North American population (20) and 47% in this group of Kenyan women who are the subject of this secondary data analysis (21).

At birth, infants are sterile, and current birthing practices are designed to optimize newborn exposure to maternal biota for which there is already immunologic protection through transplacental immunoglobulin transfer and through breastfeeding, thereby reducing the risk of infection, which is one of the leading causes of newborn deaths (7, 22). There is evidence that a newborn's gut biota is derived from the mother. Breastmilk has the bifidus factor, a product that promotes the growth of lactobacilli in the infant gut. Since BV is a state of vaginal dysbiosis characterized by a deficit in *L. genera* that form an important defense mechanism in the infant gut and other mucous membranes, we hypothesized that it may be correlated with an increase in infectious disease morbidity beyond the newborn period.

HIV-exposed infants have increased vulnerability. In the first instance, BV increases the risk of maternal HIV acquisition (16, 17). Furthermore, HIV-infected women have reduced maternal transfer of immunoglobulins against common infections to the fetus (22). This study provides an opportunity to determine whether exposure to BV further accentuates the vulnerability of HIV-exposed infants.

There is emerging evidence that child survival is closely linked to a mother's vital status; the younger a child is when a mother dies, the more likely it is that the child will die (23). Nguyen et al. documented a 35-fold higher risk of infant death in the first 6 months of life, if the mother's death was before 42 days of life (23). In the present study population, we previously reported that there was a nearly 8-fold increased risk of death when the mother died (21). The present study aimed to investigate whether there are any differences in the health of mothers and infants exposed to BV after birth. This study is a secondary analysis based on a 25-year-old dataset of the randomized trial of breastfeeding and formula-feeding among HIV-infected women, carried out to determine the risk, timing, and correlation of transmission before the availability of anti-retroviral therapy by Nduati et al. The study population is unique in the sense that the women were well-investigated for sexually transmitted infection during pregnancy, and there was a careful structured longitudinal follow-up of the babies up to 24 months of life, which has enabled the research team to carry out various analyses based on this data set. The findings of this study contribute to a better understanding of the role of BV in maternal and child health and informs policies regarding the same.

## 2. Methods

### 2.1. Study Design

This analysis is based on a retrospective cohort study design. The study methods that were used for the original study have previously been described (21). Briefly, the study was conducted in Nairobi with active enrollment from November 6, 1992 to October 7, 1997.

### 2.2. Study Population, Enrollment, Delivery, and Follow-Up

#### 2.2.1. Study Population

**Figure 1:**
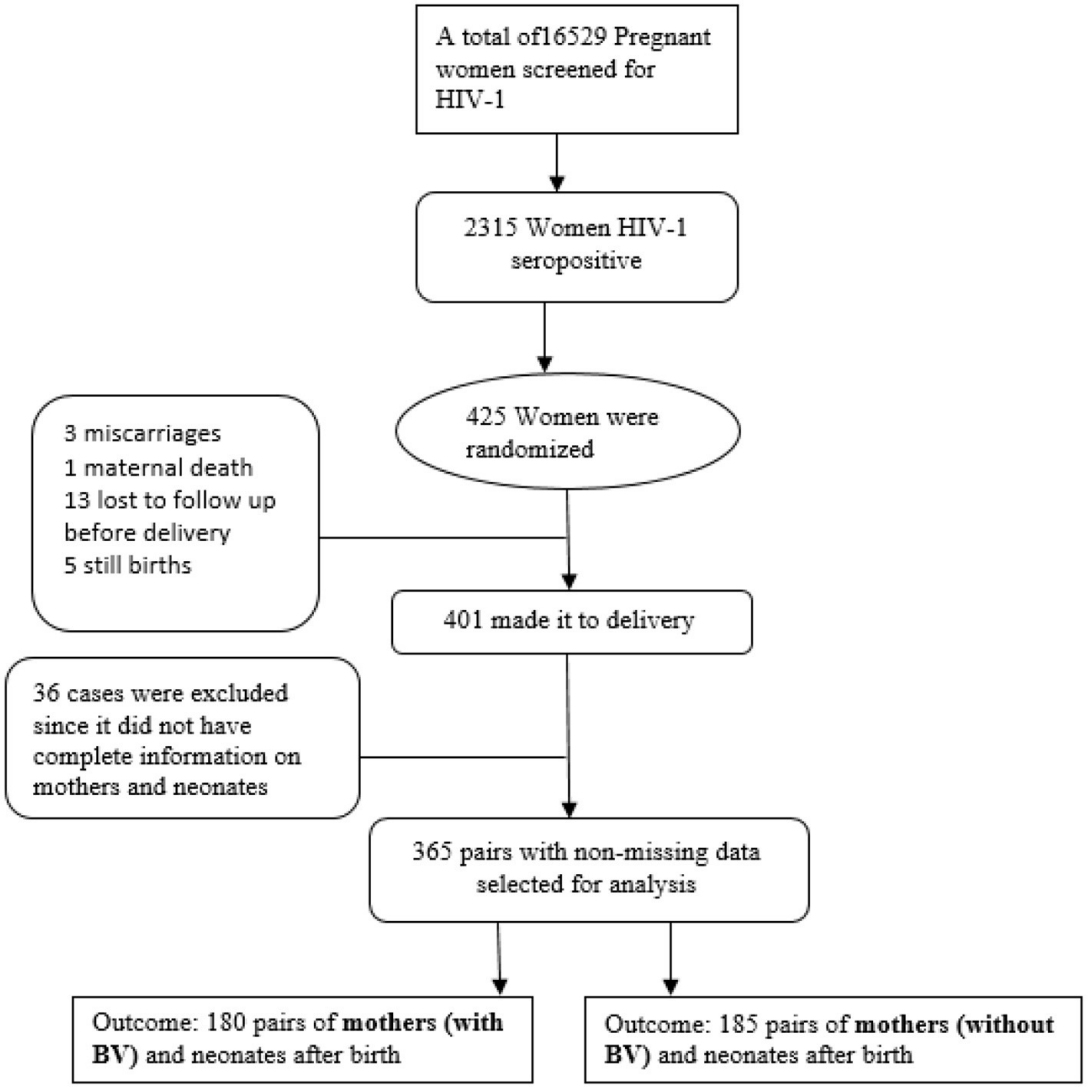
Trial flow diagram describing the recruitment and exclusion of cases.

The original study recruited 425 women, 401 of whom had live births. This study is restricted to 365 pairs who had complete matching information in terms of the BV status of the mother and requisite neonatal characteristics and included 180 women with BV and their exposed infants and 185 without BV. A total of 36 mother baby pairs were excluded because of missing information on the BV status as depicted by the flowchart shown in Figure 1. The analysis of the infant data was scaled down to 328, including 157 and 171 infants who were BV-exposed and unexposed, respectively. Thirty-seven pairs were excluded from further analysis for the following reasons: 14 babies died, and their morbidity measures were not assessed thereafter, 6 mothers were lost to follow-up, and 17 had missing data. These were finally included in the generalized estimating equation (GEE) analysis.

#### 2.2.2. Maternal Enrollment

Pregnant women attending Nairobi City Council clinics were offered HIV testing as an integrated pregnancy assessment and those who tested positive were offered participation in the study. At 32 weeks of pregnancy, after obtaining informed consent, women were subjected to a standard interview and physical examination that included a pelvic examination using a speculum to facilitate collection of vaginal and cervical secretions for microscopy and gram staining for BV, in addition to screening for sexually transmitted diseases and genital shedding of HIV. Cervical and vaginal samples were collected separately using sterile Dacron swabs. To determine the viral load and CD4—8 cell counts, 15 ml of blood was collected in purple-top vacutainers at enrollment.

#### 2.2.3. Delivery

Women were encouraged to deliver at Kenyatta National Hospital (KNH) and nurse midwives of the study team provided 24-h care to facilitate this process. At delivery, a standard form was used to collect delivery data and a cord blood sample.

#### 2.2.4. Follow-Up

The mother-baby pairs were followed up with at 6, 10, and 14 weeks, and then monthly until the child was 1 year old, and thereafter every 3 months until 24 months of age or death of the infant. Blood samples were collected at pre-determined time points during the follow-up for HIV testing.

At each visit, interviews were conducted, and information was gathered using a standard tool on any symptoms of illness since the previous encounter and during the current visit, and of any illness or hospital visits/admissions in the period since the last clinic visit. This was done regardless of whether it was a scheduled or unscheduled visit due to illness. If the participants had sought medical care elsewhere, the take-home medical records or discharge summaries were examined, and information was abstracted to ensure completeness of documentation for the morbidities. The mother-baby pairs underwent a physical examination, their clinical characteristics were documented using a standard tool and the relevant study samples were collected by the team of study doctors, which included an obstetrics registrar and three consultants, two of whom are pediatricians, and one who is a medicine-pediatrics specialist.

To capture all morbidity events, the participants had unrestricted access to care in the study clinic that operated for 4 days a week and they received careful instructions on how to navigate the hospital for other services outside clinic hours. The study participants received a standard transport reimbursement for every visit. The women were encouraged to attend the clinic from where they were enrolled for the child's follow-up, and to make sure the child received all the scheduled immunizations.

The primary intention-to-treat analysis was published in 2000, and the key findings were as follows: cumulative risk of infant infection at 24 months was 36.7% in the breastfeeding arm and 20.5% in the formula arm, and the estimated absolute risk of breastmilk transmission was 16.2 with 75% of the risk difference between the two arms of the study being achieved by 6 months (21). HIV-free survival in the breastfeeding and formula arms were 58.0 and 70.0%, respectively (*p* = 0.02). The 2-year cumulative mortality of the children was 24.4 and 20.0% in the breastfeeding and formula arms, respectively (*p* = 0.30) (21, 24). HIV-infected children have a 9-fold increased risk of death, and, of note, the incidence of diarrhea and pneumonia were identical in both arms of the study.

### 2.3. Clinical Characteristics During Follow-Up/Data Used for This Secondary Analysis

For purposes of this study, the following data were retrieved from the main dataset.

#### 2.3.1. Mothers

We abstracted data on demographic characteristics (age, education, marital status), past reproductive health (number of pregnancies, live births), baseline viral load, whether they had BV or not, and delivery-related complications (excessive bleeding, urinary tract infection, hypertension), as well as the history and duration of hospitalization.

#### 2.3.2. Newborns

We abstracted information on their status at delivery including, anthropometric measurements [length (cm), head circumference (cm), weight (g)], Apgar score at 1 and 5 min after birth, and estimated gestational age using the Dubowitz scores. Newborns with an estimated gestation of >37 completed weeks were classified as premature. Any breathing issues were classified as respiratory distress. Information was abstracted on whether the newborn had jaundice, conjunctivitis, lymphadenopathy, a skin rash, respiratory distress, or any other abnormalities after birth.

#### 2.3.3. Infant Status During Follow-Up

Data was abstracted on presence of common childhood morbidities, including but not limited to pneumonia, ear infection, blood in stool, lymphadenopathy, encephalopathy, sepsis, conjunctivitis, dehydration, wheezing, hematologic conditions, cold, otitis, fever, cough, diarrhea, thrush, vomiting, difficulty in feeding, heat rash, fungal rush, eczema/dermatitis, scabies, and mouth ulcer.

## 3. Laboratory Methods for Detection of BV

Women were categorized as having BV or not using the Nugent criterion (25) as described in the original study.

## 4. Ethical Approval

The study protocol was approved by the ethics review boards of the University of Washington and the University of Nairobi.

## 5. Statistical Analysis

Women who had BV and their exposed infants were compared to those who did not have BV. Mean values were used for characteristics with normal distribution, medians for the skewed data, and proportions for the categorical data. Effects of BV were measured and reported as a relative risk (RR) or odds ratio (OR).

To estimate the risk of BV of mothers during birth on (1) whether they needed hospital admission after facility delivery and (2) had adverse maternal condition, we estimated the RR.

We assessed the viral load data for normality using the histogram and found that it violated the bell shape required for normal distribution; therefore, we calculated the median viral load of 39,482 copies per ml. We then assessed the women with a viral load count more than the median, and less than the median, using chi-square statistics to check for statistical significance. We performed a log transformation of the viral load data to determine whether there was an association of the BV with HIV and used t-statistics to calculate the *P*-value to check for significance.

We compared BV-exposed and unexposed infants. For continuous data, we performed normality checks through standardized normal probability plots. Curves showing the bell shape of the normal distribution were considered normal data, and a *t*-test was performed, whereas curves that deviated from the assumption were considered non-normal data, and the non-parametric and Mann-Whitney *U* tests were performed to determine any associations. All tests were two-tailed. For the normal data, we reported data as the mean and standard deviation, whereas for the non-normal data, we reported data as the median and inter-quantile range.

We subsequently analyzed all the morbidity incidences ever reported on the infants between the two groups using Pearson's chi-squared test and computed the *p*-values using the Fisher's exact test. For the infant data obtained at 6 and 12 months after birth, we used the Generalized Estimating Equation (GEE) because the measures were repeated, and we had to control for the correlation. We employed the independence correlation structure, according to Hardin and Hilbe (26), with an assumption that the correlation among time points of infection is independent.

The inclusion of all the morbidities in the model was supported by the fact that some morbidities that were not significant or associated with BV were significant in the multiple logistic regression. Additionally, we adopted the suppressor effect concept proposed by Sun et al. (27) and argue that the methodological approach could show some patterns of the disease, which the conventional approaches fail to reveal.

Finally, to assess the effects of BV on survival between the two groups, we estimated the cumulative hazard using the Kaplan–Meier method. Our data met the two most important assumptions for this approach, namely: (1) censored subjects had the same risk as those remaining in the analysis; thus, censoring was non-informative; and (2) timing of exposure was known exactly; thus, infants exposed to BV were known before birth by checking their mothers' BV status. All cases of deaths from birth were included in the analysis.

All statistical analyses were performed using R version 3.6.3 (R Development Core Team, Vienna, Austria) (28). Analysis items with *P* < 0.05 were considered statistically significant.

## 6. Results

### 6.1. Demographic Characteristics of the Women

The mean age was 23.5 years (range 17–39) for the BV-exposed infants and 24.1 years (15–38) for the unexposed infants. Among BV-exposed women 27.2% had not completed the 8 years of primary education, 19.6% had completed 8 years, and 53.3% had >8 years of education compared to 35.9, 17.2, and 46.9%, respectively among the unexposed women, however these differences were not significant (*p* = 0.18). The majority of the women were married, 81% of the BV-exposed group and 73.4% of the unexposed group, and these differences were not significant (*p* = 0.18) (Table 1).

**Table 1:** Comparison of the mothers demographic and selected maternal characteristics between the two groups.

**Variable**	**Exposed**	**Unexposed**	**RR (95% CI)**	***P*-value**
**MATERNAL DEMOGRAPHICS**				
Age: Mean (Range)	23.5 (17–39)	24.1 (15–38)		0.21
Marital status: *N* (%)				
Single	28 (15.2%)	42 (21.9%)		0.18
Divorced/Separated/Widowed	7 (3.8%)	9 (4.7%)		
Married	149 (81.0%)	141 (73.4%)		
Education: *N* (%)				
<8 years	50 (27.2%)	69 (35.9%)		0.18
8 years complete	36 (19.6%)	33 (17.2%)		
>8 years	98 (53.3%)	90 (46.9%)		
Number of pregnancies: Mean (Range)	2 (1–8)	2 (1–6)		0.22
Number of live births: *N* (%)				
0	73 (39.7%)	75 (39.1%)		0.74
1 and 2	87 (47.3%)	86 (44.8%)		
>3	24 (13.0%)	31 (16.1%)		
Birth outcome: *N* (%)				
Live	168 (97.7%)	181 (98.4%)		0.31
Intra-partum deaths	2 (1.2%)	3 (1.6%)		
Still births	2 (1.2%)	0 (0%)		
Any STD: *N* (%)				
Yes	52 (28.3%)	59 (30.7%)		0.71
No	132 (71.7%)	133 (69.3%)		
**MATERNAL CHARACTERISTICS**				
Log viral load per milliliters: Mean (SD)	10.64 (1.79)	10.37 (1.88)		0.19
Viral load >39, 482: *N* (%)				
Yes	79 (53.3%)	76 (46.3%)	1.15 (0.92–1.44)	0.25
No	69 (46.7%)	88 (53.7%)		
Admitted after birth?: *N* (%)				
Yes	32 (19.3%)	16 (9.7%)	1.99 (1.14–3.48)	0.02
No	134 (80.7%)	149 (90.3%)		
Adverse maternal conditions?: *N* (%)				
Yes	16 (9.7%)	7 (4%)	2.45 (1.04–5.81)	0.04
No	149 (90.3%)	170 (96%)		

### 6.2. Clinical Characteristics of Women Participants

The women had a mean of two pregnancies and a median of one live birth. Among the BV-exposed women 39.7, 47.3, and 13.0%, 0, 1–2, and > 3 previous live birth vs. 39.1, 44.8, and 16.1% among unexposed women. These differences were not significant (*p* = 0.74). Furthermore, 28.3% of the BV-exposed women reported having had an STD compared to 30.7% of the unexposed women, showing a non-significant difference (*p* = 0.71) (Table 1).

A total of 79 (53%) BV-exposed women and 76 (46%) unexposed women had a high viral load, higher than the calculated median (39482); the association of a higher viral load with BV was not statistically significant (RR = 1.15, CI = 0.92, 1.44, *p* = 0.25). The mean log viral load was 10.64 and 10.37 copies per ml for women afflicted with BV and those who were not, respectively, but results were not significant (*p* = 0.19) as shown in Table 1.

### 6.3. Post-delivery Maternal Morbidity

Maternal conditions were reported by 23 women, including 16 (10%) of the 165 BV-exposed women and seven (4%) of the 177 unexposed women. The risk for an adverse maternal condition was significantly associated with BV RR = 2.45 [(95% CI 1.04, 5.81) *p* = 0.04] (Table 1).

### 6.4. Maternal Hospital Admission

Among 331 women who delivered in hospital, 48 (15%) required extended hospital admission, 19.3% were BV exposed, and 9.7% were unexposed to BV, RR = 1.99 [(95% CI 1.14–3.48), *P* = 0.02] as shown in Table 1. Forty-two women required admission for more than 1 day, including 21 (12.6%) of the 166 BV-exposed women and 15 (9.1%) of the 165 unexposed women; the difference was not significant (*p* = 0.3). BV-exposed women had a longer duration of admission compared to unexposed women, a higher maximum admission of 21 days against 16 for the unexposed. The mean hospital admission duration was 1.3 ± 3.2 days among women with BV and 0.7 ± 2.7 days among women with BV (*p* = 0.91).

### 6.5. Characteristics of the Neonates

Eighty-one (46.5%) of the 174 BV-exposed infants and 99 (55.5%) of the 179 unexposed infants, respectively, were male. The mean head circumference in both groups was 35.2 cm. BV-exposed babies were lighter than the unexposed infants, 3,096 vs. 3,196 g, an important trend on statistical testing (*p* = 0.08). In addition, five (3%) of the infants exposed to BV had a LBW (<2,500 g) compared to one (1%) in the unexposed infants (*p* = 0.2). BV-exposed and unexposed babies were of comparable length, and gestation. In addition, BV-exposed and unexposed neonates had comparable prevalence of neonatal morbidities that included jaundice (10.1 vs. 8.5%, *p* = 0.76), conjunctivitis (7.1 vs. 5.2%, *p* = 0.61 lymphadenopathy (6.5 vs. 9.1%, *p* = 0.76), respiratory distress (1.2% each, *p* = 0.9), and skin rash (11.8 vs. 12.6%, *p* = 0.94) (Table 2).

**Table 2:** Neonatal characteristics and morbidities after birth.

	**Exposed**	**Unexposed**		
**Variable**	**Mean (SD)/*N* (%)**	**Mean (SD)/*N* (%)**	**RR (95%CI)**	***P*-value**
**NEONATAL CHARACTERISTICS**				
Sex: *N* (%)				
Female	93 (53.5%)	79 (44.5%)		0.11
Male	81 (46.5%)	99 (55.5%)		
Maturity: Mean (SD)	39.5 (SD: 2.35)	40.0 (SD: 2.03)		0.26
Head circumference: Mean (SD)	35.2 (SD: 1.49)	35.3 (SD: 1.59)		0.88
Birth weight (in grams): Mean (SD)	3096 (SD: 547)	3196 (SD: 498)		0.08
Length: Mean (SD)	48.1 (SD: 4.3)	48.6 (SD: 2.45)		0.30
>37 completed gestation weeks: *N* (%)				
Yes	103 (92.8 %)	114 (95 %)	0.98 (0.92–1.04)	0.67
No	8 (7.2 %)	6 (5 %)		
Low birth weight: *N* (%)				
Yes	5 (3%)	1 (1%)	5.33 (0.63–45.11)	0.19
No	164 (97%)	179 (99%)		
Dead: *N* (%)				
Yes	9 (5%)	5 (3%)	1.85 (0.63–5.41)	0.39
No	171 (95%)	180 (97%)		
**NEONATAL MORBIDITIES**				
Jaundice: *N* (%)				
Yes	17 (10.1%)	15 (8.5%)	1.18 (0.61–2.29)	0.76
No	152(89.9%)	161(91.5%)		
Conjunctivitis: N (%)				
Yes	12 (7.1%)	9 (5.2%)	1.36 (0.59–3.16)	0.61
No	158 (92.9%)	165 (94.8%)		
Lymphadenophathy: *N* (%)				
Yes	11 (6.5%)	15 (9.1%)	0.72 (0.34–1.52)	0.51
No	158 (93.5%)	151 (90.9%)		
Respiratory distress: *N* (%)				
Yes	2 (1.2%)	2 (1.2%)	1.01 (0.14–7.10)	0.99
No	168 (98.8%)	170 (98.8%)		
Skin rash: *N* (%)				
Yes	20 (11.8%)	22 (12.6%)	0.94 (0.53–1.65)	0.94
No	150 (88.2%)	153 (87.4%)		
Other abnormalities after birth: *N* (%)				
Yes	5 (2.9%)	4 (2.3%)	1.28 (0.34–4.68)	0.97
No	165 (97.1%)	170 (97.7%)		

### 6.6. Characteristics of the Infants

We first evaluated the “ever had” morbidity incidence, in which any infant who recorded any incidence was analyzed. In the test of association between BV and the various morbidities, only hepatomegaly and having a cold showed statistical significance. No other morbidities assessed showed any association with BV at *P* < 0.05. Using GEE to control for repeated events, the odds of morbidity at 6 and 12 months of infants exposed and unexposed to BV are shown in Table 3.

**Table 3:** Predictors of bacterial vaginosis at 6 and 12 months with corresponding 95% Confidence Intervals (CI) and *p*-values.

		**6 months**		**12 months**	
		**OR (95% CI)**	***P*-value**	**OR (95% CI)**	***P*-value**
	Pneumonia	1.14 (0.75–1.72)	0.54	1.20 (0.88–1.65)	0.25
	Ear infection	0.55 (0.18–1.54)	0.27	0.63 (0.33–1.17)	0.14
Respiratory	Wheezing	0.67 (0.34–1.27)	0.22	0.87 (0.59–1.27)	0.47
infections	Cold	1.09 (0.88–1.35)	0.44	0.99 (0.84–1.16)	0.88
	Otitis	1.10 (0.60–2.01)	0.76	1.10 (0.79–1.55)	0.57
	Cough	0.97 (0.77–1.23)	0.81	0.93 (0.78–1.10)	0.39
	Stool with blood	3.08 (1.11–10.00)	0.04	1.19 (0.67–2.12)	0.56
Gastrointestinal	Dehydration	2.94 (1.44–6.37)	0.01	1.81 (1.05–3.19)	0.03
infections	Diarrhea	0.72 (0.43–1.22)	0.23	0.64 (0.45–0.89)	0.01
	Vomiting	1.64 (1.06–2.56)	0.03	1.39 (1.01–1.92)	0.04
	Mouth ulcers	12.8 (2.27–241.21)	0.02	2.34 (1.00–6.03)	0.06
	Lymphadenopathy	0.74 (0.55–0.99)	0.04	0.65 (0.52–0.82)	0.01
	Encephalopathy	0.55 (0.07–3.57)	0.53	0.51 (0.07–2.74)	0.45
	Sepsis	1.27 (0.55–2.96)	0.57	1.18 (0.53–2.65)	0.68
	Conjunctivitis	1.32 (0.84–2.08)	0.24	1.41 (0.95–2.10)	0.09
	Difficulty feeding	0.74 (0.49–1.12)	0.16	0.78 (0.63–0.97)	0.02
Other	Heat rash	0.79 (0.55–1.13)	0.2	0.75 (0.56–1.01)	0.06
infections	Fungal rash	1.04 (0.62–1.77)	0.87	1.15 (0.75–1.78)	0.51
	Eczema/dermatitis	0.95 (0.76–1.19)	0.67	0.87 (0.73–1.04)	0.13
	Scabies	0.78 (0.37–1.62)	0.5	1.04 (0.69–1.55)	0.86
	Hepatomegaly	0.47 (0.19–1.05)	0.08	0.56 (0.32–0.94)	0.03
	Fever	0.75 (0.57–0.99)	0.04	0.89 (0.73–1.07)	0.22
	Thrush	0.94 (0.64–1.37)	0.74	0.92 (0.67–1.28)	0.63

At 6 months of age, infants exposed to BV had significantly higher odds of reporting gastrointestinal symptoms of illness, including symptoms of passing bloody stool [OR 3.08 (1.11, 10.00), *p* = 0.04] and vomiting OR 1.64 (1.06, 2.56), *p* = 0.03], signs of dehydration [OR 2.94 (1.44, 6.37), *p* = 0.01], and mouth ulcers [OR 12.8 (2.27, 241.21), *p* = 0.02]. Babies exposed to BV were less likely to have lymphadenopathy [OR 0.74 (0.55, 0.99), *p* = 0.04] and fever [OR 0.75 (0.57, 0.99), *p* = 0.04] (Table 3).

At 12 months, there was a substantial decrease in the number of gastro-intestinal morbidities. Although, the exposed group had higher odds for the same, the trends for dehydration [OR 1.81 (1.05, 3.19), *p* = 0.03] and vomiting [OR 1.39 (1.01, 1.92), *p* = 0.04)] decreased. Babies exposed to BV were less likely to have lymphadenopathy [OR 0.65 (0.52, 0.82), *p* = 0.01], difficulty in feeding [OR 0.78 (0.63, 0.97)], and hepatomegaly [OR 0.56 (0.32, 0.94), *p* = 0.03]. The other characteristics assessed were not significant at the 0.05 level (Table 3).

BV-exposed and unexposed infants had similar incidence of conditions affecting the respiratory and other systems at 6 and 12 months.

## 7. Mortality in the First 12 months of Life

**Figure 2:**
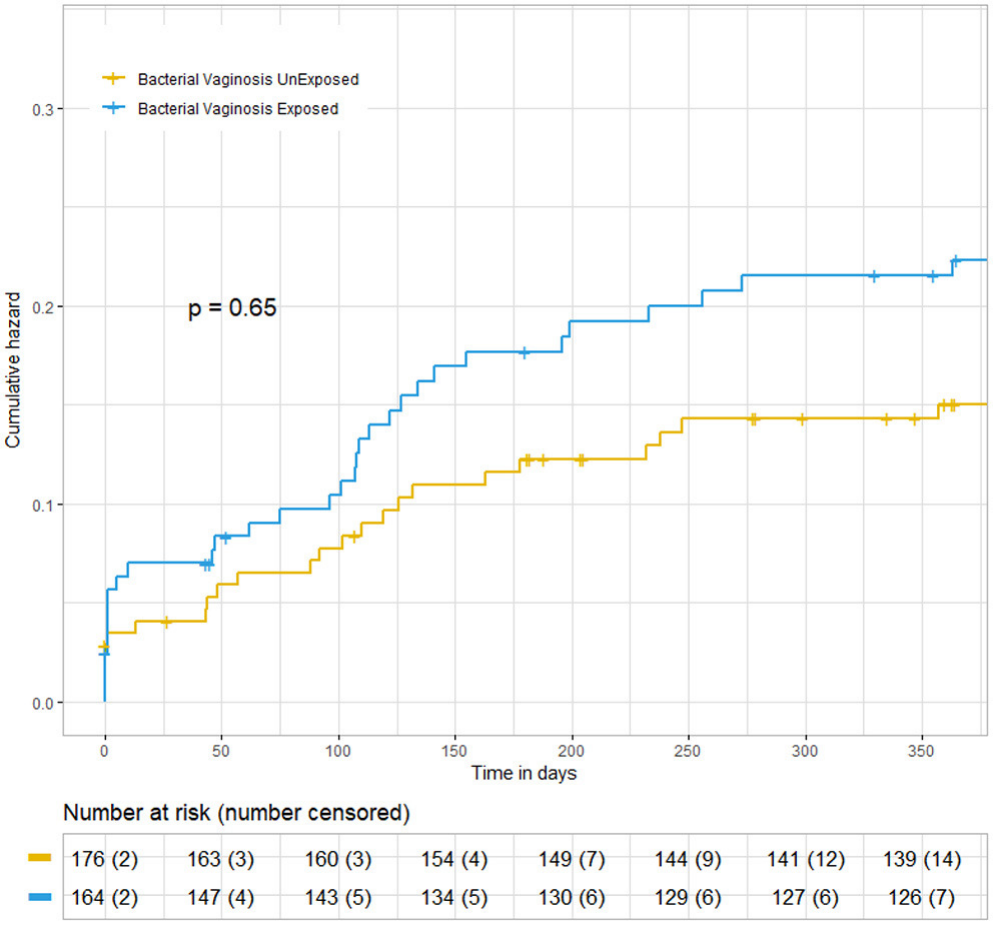
Kaplan–Meier analysis of infant mortality over 12 months between infants with and without maternal exposure to bacterial vaginosis.

In the BV-exposed pregnancies, there were two still births and two intrapartum deaths, in contrast to zero still births and three intrapartum deaths. Over the first year, there were 14 deaths in the first year, (9 [5%]) of 180 in the exposed group, and (5 [2.8%]) in the 185 unexposed group, showing a risk difference of 2.2%. The relative risk of death in the exposed group was RR = 1.85 [(95% CI 0.63–5.41), *p* = 0.39]. We compared the survival between infants whose mothers were exposed to BV and those whose mothers were not, using the KM (Figure 2). The graph showed a trend of higher mortalities in the BV-exposed group. The difference was not statistically significant (*p* = 0.65).

## 8. Discussion

In this study, we examined the association of maternal BV with a mothers' condition and infants' morbidities. Our analysis showed an increase in the frequency of maternal hospital admissions among women with BV compared to non-exposed subjects. In our literature review, we did not find any publication that directly reports on maternal perinatal adverse outcome associated with BV, and reports have universally focused on fetal/neonatal outcome. The closest article was a systematic review on the use of probiotics to improve maternal microbiota, which demonstrated a reduction in gestational diabetes and better blood sugar control. Prolonged hospital admission has a cost implication and increases the risk of nosocomial infections (29).

Exposure to BV did not show any association with any neonatal morbidities, as previously reported (21). However, the lower mean birth weight in the exposed group in this study was in line with the findings reported by others (30–32). Women who were diagnosed with BV were provided treatment in the third trimester of pregnancy, and this may have attenuated the differences between BV-exposed and unexposed women.

The most novel observation from this study was the link between BV and morbidities among infants at 6 and 12 months after birth. The children exposed to BV had significantly more symptoms of gastrointestinal diseases. Additionally, there was increased mortality among infants exposed to BV, although, the latter did not achieve statistical significance. These observations support our hypothesis that babies exposed to BV have Lactobacillus-deficient biota characterizing BV, making them more vulnerable to gastrointestinal infections (33). It is prudent to note that these effects were more commonly observed in the first 6 months of life and decreased as the baby grew older. This is supported by our previous analysis of this data that reported a decrease in morbidities with time (34).

Fouhy et al. in the 2012 comprehensive review of the composition of early intestinal microbiota observe that the gut is sterile at birth and is progressively colonized by bacteria achieving an adult like microbiome by the age of 2 (35). The initial colonization of the gut influences the subsequent development of the immune system, including the gut associated lymphoid tissues and that gut biota play a role in the regulation of the immune system. It is further postulated that an altered gut biota composition may predispose the infant to infections and allergic reactions. In this study we show that BV-exposed babies have increased gastrointestinal infections. Consistent with the concept of a maturing gut microbiota, the incidence of the infections diminished as the infants advanced in age.

Studies contrasting babies born vaginally with those born by caesarian section show that the gut is first colonized with the mother's vaginal and fecal microbes, which quickly helps to establish the dominant number of lactobacillus within a few hours of delivery. In contrast, Cesarean babies are first colonized with maternal skin microbes with a predominance of staphylococci. It is reasonable to assume that BV-exposed infants were colonized with bacteria typical of vaginal dysbiosis (35).

A number of studies have looked at the long-term impact of being born vaginally vs. Cesarean, therefore acquiring different gut biota, and found worse outcomes in allergy and metabolic conditions, such as asthma and obesity among those born by Cesarean (35). We have not found any studies reporting on the gut biota of babies born to women with vaginal dysbiosis. BV is best known for its role in increasing risk of prematurity. Based on the findings of this study, there is a need to further evaluate its contribution to infant infections and mortality.

Unexposed subjects had higher odds of manifesting lymphadenopathy, hepatomegaly, difficulty feeding, and fever, and may reflect reverse causality. The BV-exposed children may have had greater access to antimicrobial therapy, which would have treated a variety of illness beyond what they were reported to have, while the unexposed children went on to have a full expression of their illness.

There has not been any published literature linking child mortality directly to BV. However, there is an indirect link through the related adverse pregnancy outcome, including pre-term birth, prematurity, and pregnancy complications, which are factors directly related to neonatal and infant mortality (36). Our Kaplan–Meier analysis, through the cumulative hazard plot, showed a non-significant difference (*p* = 0.65); however, the graph showed a trend toward a higher mortality rate in the BV-exposed group. Therefore, this means that the risk of mortality still exists among infants whose mothers are exposed to BV. Our study was not powered to fully evaluate this. Future studies regarding BV and mortality are needed to confirm our results and to elucidate the underlying associations.

We believe the results of our study are credible. The diagnosis of BV was made using the Nugent method, the gold standard, and therefore, there is clarity on the infants' exposure. The technologist who examined the slides was part of the team that developed the Nugent method for diagnosing BV. We used the Nugent method to diagnose BV, which has the advantage of having a standardized scoring system, and therefore, reduces variability in reporting. The alternative method would have been to culture the bacteria, but at the time, there was limited microbiology culture capacity, and diagnosis of BV was not the primary endpoint of the study. Since then, the development of the field of genomics has expanded on opportunities to describe the microbiota, and in the process, has expanded the spectrum of bacteria identified to be part of the human microbiome (10, 13). Studies that have evaluated these newer techniques along with the newer genomic studies have found congruent study findings (35).

The infants were seen frequently for both scheduled and unscheduled visits as needed, and a standard tool was used to collect the data, and therefore, we are confident regarding the documentation about the presence or absence of a specific morbidity. The clinics were run by very qualified staff; thus, the data collected was of high quality, there were few cases with missing data, and therefore, the results are generalizable. For example, of the 401 mother and infant pairs in the original study, the prevalence of BV as reported by Nduati et al. (21) was 47%. Subsequently, on the final pairs included in our analysis, the prevalence of BV was calculated as 48% (159/328). This is consistent with the findings of other studies that reported the prevalence of BV in Kenya to be between 30 and 50% (37). In terms of methodology, the use of GEE is based on its unique property of handling repeated measures.

In this study, the syndromic treatment of women with abnormal vaginal discharge, which covers for BV, and prompts the treatment of children's illness, means that effects of BV are attenuated toward null. As observed earlier, participants received transport reimbursement and the research clinic provided a safe space when HIV was still new and very stigmatized. Both BV-exposed and unexposed babies had a median number of eight visits during the first year of life. With this, we were not able to establish the correct frequency of hospital visits due to genuine morbidities.

## 9. Conclusions and Future Research

This paper reported many findings that are consistent with those reported in the literature and has added further knowledge in the area of BV. While previous studies have focused only on the effects of BV on infants, our study explored the effects of BV on both the mother and baby in the context of HIV. This has an implication for infants who are more vulnerable to several infections due to a compromised immune status. Our study has shown independence in terms of association of BV with HIV and recommends for BV to be researched alone. Future research is recommended to gain deeper insights into the predictors of BV.

## Data Availability Statement

The datasets presented in this study can be found in online repositories. The names of the repository/repositories and accession number(s) can be found at: https://osf.io/yxbhg/files/.

## Ethics Statement

The studies involving human participants were reviewed and approved by University of Nairobi and Washington Institution Review Board. Written informed consent to participate in this study was provided by the participants' legal guardian/next of kin.

## Author Contributions

All authors listed have made a substantial, direct and intellectual contribution to the work, and approved it for publication.

## Conflict of Interest

The authors declare that the research was conducted in the absence of any commercial or financial relationships that could be construed as a potential conflict of interest.
